# Peri-implant diseases triggered by oral microdysbiosis: pathogenesis and precision intervention strategies

**DOI:** 10.3389/fmicb.2025.1639095

**Published:** 2025-08-07

**Authors:** Gang Chen, Xin Zhao, Bin Yang, Hongzheng Gu

**Affiliations:** ^1^Department of Implant and Restoration, Zhenjiang Stomatological Hospital, Zhenjiang, China; ^2^Central Laboratory of Zhenjiang Stomatological Hospital, Zhenjiang, China; ^3^Expert Consultation Center of Zhenjiang Stomatological Hospital, Zhenjiang, China

**Keywords:** peri-implant mucositis, peri-implantitis, dysbiosis, oral pathogenic bacteria, intervention and treatment strategies

## Abstract

Peri-implant disease is a chronic infection-induced inflammation condition affecting tissues around dental implants, categorized into peri-implant mucositis and peri-implantitis. Oral microbial dysbiosis plays an important role in this disease. Currently, researchers face three challenges in establishing the pathogenic link between peri-implant disease and microdysbiosis: (1) elucidating the underlying molecular mechanisms; (2) Clarifying causal links between host and microbiome; and (3) Identifying secondary microbial changes during disease progression. In this review, we systematically classify dysbiosis from a conceptual perspective and outline the immunological associations within each category. We further elaborate on the causes of bacterial dysbiosis and analyze its potential implications for clinical treatment strategies. At the molecular level, understanding the origins, intrinsic and environmental regulatory mechanisms, and downstream effects may be conducive to develop microbiome targeted therapies. This research direction is of great significance for promoting precision medicine in peri-implant disease.

## 1 Introduction

With the advancement of time and technology, dental implants have become a safer and more effective means of replacing missing teeth and restoring mastication, occlusion, and aesthetic function than traditional methods of restoring missing teeth (Cui et al., 2025). However, a concomitant problem is the increasing incidence of peri-implant disease year after year, which has attracted the attention of a wide range of dentists and some social repercussions (Derks and Tomasi, 2015; Sun et al., 2023). Studies have shown that the prevalence of peri-implant disease ranges from 28 to 56% in the patient population and from 12 to 43% in implants placed (Gonzalez Regueiro et al., 2021; Chan et al., 2023), reaching 43% in Europe, South America and North America (Fu and Wang, 2020). The morphologic design of the implant, surface process treatment, material selection, and implantation technique of the practitioner can directly affect the incidence of peri-implant disease (Laleman and Lambert, 2023; Schwarz and Ramanauskaite, 2022). However, investigations have shown that factors of poor peri-implant health management occupy a higher percentage, such as poor oral hygiene, poor dietary habits, and smoking (Rahnama-Hezavah et al., 2023; Ball and Darby, 2022; Amerio et al., 2025; Zhang et al., 2022; Dereka et al., 2022). In addition, some studies have shown that genetic polymorphisms are potential risk factors for peri-implantitis (Dereka et al., 2022; Cardoso et al., 2024).

Peri-implant diseases mainly include peri-implant mucositis and peri-implantitis, which are inflammatory damage that are in the soft and hard tissues around the implant, respectively (Ball and Darby, 2022; Ramanauskaite and Schwarz, 2024). If peri-implant diseases are not effectively treated, they can eventually lead to bone resorption and implant loosening (Berglundh et al., 2018; Alterman and Casap, 2025). Plaque biofilm is the initiator of peri-implant disease, and its attachment to the implant surface leads to the development of inflammation, induces an excessive immune response, and ultimately destroys osseointegration (Roccuzzo et al., 2023; Enteghad et al., 2024; Rahman et al., 2022). However, plaque biofilms are almost naturally present on tooth surfaces, and data from the Human Microbiome Project (HMP) show that about 700 species aggregate to form highly organized biofilms that are relatively stable in structure and perform their functions in an orderly manner (Baseri et al., 2020). Therefore, under normal conditions, the plaque biofilm, as a complex, orderly, harmonious symbiosis, internal and external communication tends to be stable, and will not easily produce pathogenicity; and when oral microecological disorders lead to dysbiosis of the oral flora, the internal balance of the plaque biofilm is disrupted, followed by a chain reaction.

The oral cavity as an important gateway for communication between the human body and the outside world, stable microecology is essential for human health (Baker et al., 2023; Lin et al., 2024). The colonization of pathogens on the implant surface not only triggers peri-implant diseases, but may even progress to systemic inflammation, which is life-threatening (Radaelli et al., 2021). In this review, we systematically review the current status of peri-implant disease dysbiosis and its immune-mediated mechanisms from a microecological perspective, and summarize the relevant therapeutic strategies.

## 2 Methods

To systematically review pathogenesis and precision intervention strategies of peri-implant diseases triggered by oral microdysbiosis. Sources are searched in PubMed between January 1, 1998 and March 30, 2025. The search terms included “peri-implant disease,” “peri-implant mucositis,” “peri-implantitis,” “oral microbiota,” “oral microbiome,” “oral pathogenic bacteria,” “socransky complex,” “microecological disorders” OR “microdysbiosis” OR “microecological imbalance,” “oral dysbiosis,” “oral immune mechanisms,” “oral microbial therapy,” “clinical therapeutic strategies for peri-implant diseases.”

Abstracts of all citations were reviewed by a single author and rated for inclusion for peri-implant triggered by oral microdysbiosis. Full articles were retrieved if oral microdysbiosis was discussed. Exclusion criteria included case reports or case series, duplicate reports or trials. Finally, we selected 114 articles for narrative review, of which 7 high-quality articles were used for analysis of bacterial content.

## 3 Observations and discussions

### 3.1 Microdysbiosis and oral dysbiosis

Diversity, stability and resistance, and resilience are key concepts used to characterize microbial systems, describing their species abundance, susceptibility to perturbation, and capacity to recover to a pre-perturbation state (Fassarella et al., 2021; Levy et al., 2017). The human micro-ecosystem is self-regulating; However, when exposed to endogenous or exogenous disturbances that exceed its limits, the equilibrium-both within the microbiota and between the microbiota and the host is disrupted. This shift transforms the physiological microbial assemblage into a pathological state a phenomenon termed microdysbiosis (Levy et al., 2017). Microdysbiosis is not merely a statistically significant alteration in microbial composition but also a functionally relevant state that influences disease etiology, diagnosis and treatment. It arises from complex interactions among microbiota, environmental factors, and host conditions, with dysbiosis representing a core manifestation of this imbalance.

Oral dysbiosis is usually characterized by one or more of the following non-mutually exclusive features (Haran and Mccormick, 2021):

1. Proliferation of pathogenic commensal bacteria-bacteria that are normally present in low abundance but can proliferate under pathological conditions (e.g., *Porphyromonas gingivalis*, *Tannerella forsythia*, *Treponema denticola*, *Fusobacterium nucleatum*).
2. Deficiency of normal commensal bacteria-In contrast to the overgrowth of pathogenic commensal bacteria, a reduction in the number or absence of normally present or colonizing members of the microbiota often also results in dysbiosis, which may be caused by factors such as inappropriate use of antibiotics or systemic illnesses.
3. Loss of flora diversity-Restoration of the vanished bacteria and their metabolites has the potential to reverse dysbiosis-associated phenotype.

### 3.2 Oral dysbiosis in peri-implant disease

It was observed that the healthy peri-implant mucosa forms a ring-like soft tissue seal that closely adapts to the implant surface, which extends from the oral epithelium, with scattered distribution of inflammatory cells inside, constituting a non-keratinized barrier. Notably, the presence of appropriate number of inflammatory cells reflects the epithelial barrier’s role in defending against external bacterial invasion (Jung et al., 2022). Local dysbiosis disrupts the epithelial barrier function. The plaque biofilm continues to attach and thicken, eventually leading to the progression of peri-implant mucositis. Clinically, this condition manifests as an inflammatory lesion in the peri-implant mucosa without marginal bone loss (Lindhe and Meyle, 2008; Zitzmann and Berglundh, 2008). During the gradual progression of inflammation, peri-implant bone loss begins, pockets deepen, localized hypoxic zones emerge. Concurrently, a complex peri-implant biofilm community dominated by gram-negative anaerobic bacteria evolves, marking the transition to peri-implantitis (de Campos Kajimoto et al., 2024). At this stage, the microbial composition exhibits greater diversity and the pathogenic flora becomes more structurally complex compared to both healthy sites and those with peri-implant mucositis (Padial-Molina et al., 2024).

Peri-implant disease is characterized by microbial dysbiosis where pathogenic bacteria become dominate, and inflammatory damage spreads from local to external. S S Socransky et al. collected a large number of subgingival plaque samples from patients with periodontitis, measured and categorized the bacteria into clusters, and proposed an authoritative subgingival plaque microbial complex, the Socransky complex, which is of great significance for medical research, education, and clinical practice (Socransky et al., 1998). With the increased awareness of peri-implant diseases, numerous scholars have found that periodontal inflammatory diseases are similar to peri-implant diseases in many ways and have studied peri-implant diseases along the Socransky complex. The red and orange bacterial complexes commonly found in periodontitis in the Socransky complex overlap with most of these pathogenic bacteria, including *P. gingivalis*, *T. forsythia*, *T. denticola*, *F. nucleatum*, *Prevotella intermedia*, *Staphylococcus aureus*, *Streptococcus* spp. and *Actinobacillus* spp. (Kensara et al., 2024). After revisiting and expanding the Socransky complex, Fernandes et al. proposed the GF-MoR complex and found that important species in peri-implant mucositis included *Prevotella* spp., *P. gingivalis*, *T. forsythia*, *T. denticola*, *Actinobacillus actinomycetemcomitans*, and *F. nucleatum*, all of which are Gram-negative, with a relatively small percentage of other species (Fernandes et al., 2024). Further evidence from Jia et al.’s symbiotic network analysis identified *Fretibacterium fastidiosum* as more abundant in peri-implantitis sites compared to healthy or mucositis sites, suggesting its potential as one of the markers of peri-implantitis (Jia et al., 2024). Other studies have reported unique peri-implant pathogens, e.g., *Fretibacterium fastidiosum*, *Filifactor alocis*, *Monilia albican*, herpes simplex virus type I, human herpesvirus 4, etc.

Similar to gingivitis and periodontitis, red-complex bacteria are the most widespread and closely associated with peri-implant disease, particularly *P. gingivalis*, *T. forsythia*, and *T. denticola*. *P. gingivalis* is very highly abundant at peri-implant disease sites and plays a major role in the pathogenesis of peri-implantitis. This bacterium exhibits obvious virulence, producing proteases, capsules, as well as causative factors such as lipopolysaccharide and gingipains, which significantly disrupt tissue barriers and evade host immune responses (Mariam et al., 2024; How et al., 2016). High levels of IL-1β, IL-8, IL-6, monocyte chemotactic proteins, and matrix metalloproteinase-1 can be immediately identified in *P. gingivalis*-infected individuals, suggesting that this genus is responsible for the early inflammation and tissue damage around implants (Irshad et al., 2013). Furthermore, the coexistence of *P. gingivalis* with other bacteria magnifies the effects of the inflammation and causes more damage (Lamont et al., 2018; Makkawi et al., 2017). These findings have important clinical implications, as the severity of peri-implantitis can be measured by measuring the levels of *P. gingivalis* and IL-8 and IL-1β in the peri-implant gingival crevicular fluid (Săndulescu et al., 2023). Like *P. gingivalis*, *T. forsythia*, as another core Red Complex Bacteria, is frequently detected in peri-implantitis (Sanz-Martin et al., 2017). Studies suggest that its matrix adhesion capacity cultured in titanium powder is significantly enhanced compared to dentin, explaining its susceptibility to cause peri-implantitis (Eick et al., 2017). In peri-implant mucositis, *T. forsythia* employs its glycosylated surface antigen-BspA to invade oral epithelium and disrupt immune barriers (Schäffer and Andrukhov, 2024). *T. denticola* is a Gram-negative, anaerobic spirochete that has been consistently identified as a major pathogen in peri-implantitis microbiomes. Metagenomic and 16S rRNA sequencing studies reveal its high abundance in peri-implantitis biofilms, often co-occurring with other periodontal pathogens such as *Porphyromonas gingivalis* and *Tannerella forsythia*. *T. denticola* is significantly correlated with peri-implantitis clinical parameters, including probing depth (PPD), bleeding on probing (BOP), and radiographic bone loss (RBL) (Song et al., 2024). Studies report its elevated abundance in peri-implantitis sites compared to healthy controls, irrespective of periodontal status, suggesting its independence from secondary conditions (Kensara et al., 2021).

Orange-complex bacteria play an important role in the progression of peri-implant disease and usually serving as sentinel bacteria for red complex bacteria. Key representatives include *F. nucleatum*, *Prevotella intermedia* and *P. micra*. The morphology of *F. nucleatum* allows it to function as a “bridging bacterium” in biofilms. It secretes multiple lectins that mediate intra- and intergeneric bacterial adhesion, thereby promoting co-polymerization, and establishing strong junctions between other bacteria. This mechanism facilitates adhesion, colonization, and biofilm formation (Kolenbrander, 2011; Shi et al., 2022). *F. nucleatum* is present in peri-implant mucositis and plays a role in early disease progression; Its detection rate is high in patients with active inflammation progression and severe inflammatory destruction, underscoring its role in disease progression (Shi et al., 2022; Wang et al., 2021). *Prevotella intermedia* is a Gram-negative anaerobic bacterium that is more common in the oral microbiota, with higher abundance in subgingival flora of patients with peri-implant disease compared to healthy sites. *Prevotella intermedia* produces a variety of virulence factors that drive its colonization. Therefore, it has a strong invasion and exceptional intra-host adaptation abilities through the secretion of multiple substances, including adhesins, proteases, hemagglutinins, hemolysins, lipopolysaccharides, and capsular antigens, which subverts host immune defenses, ultimately leading to tissue destruction (Săndulescu et al., 2023). The detection of *Prevotella intermedia* in both periodontal and peri-implant diseases highlights the need for targeted antimicrobial therapies. Its presence in healthy implants (5.56%) suggests a latent pathogenic potential, necessitating vigilant monitoring in high-risk patients (Fernandes et al., 2024). Future research should explore its role in systemic inflammation and antibiotic resistance patterns. *P. micra* is associated with the early development of peri-implantitis, One comparative study reveals that *P. micra* is only detectable in peri-implantitis group but absent in periodontitis cases, suggesting its association with early development of peri-implantitis (Koyanagi et al., 2013).

The yellow-complex bacteria, represented by *Streptococcus* spp., are generally associated with healthy periodontal and peri-implant environments. However, some studies have shown their presence in large numbers within peri-implantitis environments (Chun Giok and Menon, 2023). Although generally considered beneficial, certain Streptococcus species can play an unfavorable role under specific conditions. Emerging evidence suggests that yellow-complex bacteria may provide a protective layer for periodontal pathogens (Avila et al., 2009; Gordon, 2020). *Streptococcus sanguinis* demonstrates its role through several mechanisms: (1) its pili protein PilC can bind to *α*-amylase in saliva and promote the formation of biofilm; (2) it may stimulate gingival epithelial cells to produce IL-8 and *β*-defensins, which may protect the tissue from periodontitis-associated pathogens; and (3) it elicits weaker host immune responses compared to *Porphyromonas gingivalis* or *Fusobacterium nucleatum*, which may be one of the mechanisms that benefit the host (Zhu et al., 2018; São-José et al., 2022). The bidirectional role of yellow complex bacteria has been continuously explored. Detailed exploration of these mechanisms of action may enrich peri-implant disease prevention and treatment strategies.

Green, purple, and blue complex bacteria are generally associated with healthy periodontal and peri-implant environments. The GF-MoR complex proposed by Fernandes et al. reveal that even though those three complexes bacteria constitute a lower percentage of the microbiota compared to red and orange complexes, they can still be detected in lesions; Their presence suggests an active host defense against pathogenic flora, but the exact mechanism and effect is not clear (Fernandes et al., 2024). This observation motivates investigation into whether these three complexes can competitively colonize lesion sites in a suitable ratio or even expel the pathogenic bacterial flora by decreasing their numbers, ultimately attenuating peri-implant inflammatory damage and promoting tissue recovery.

**Figure 1:**
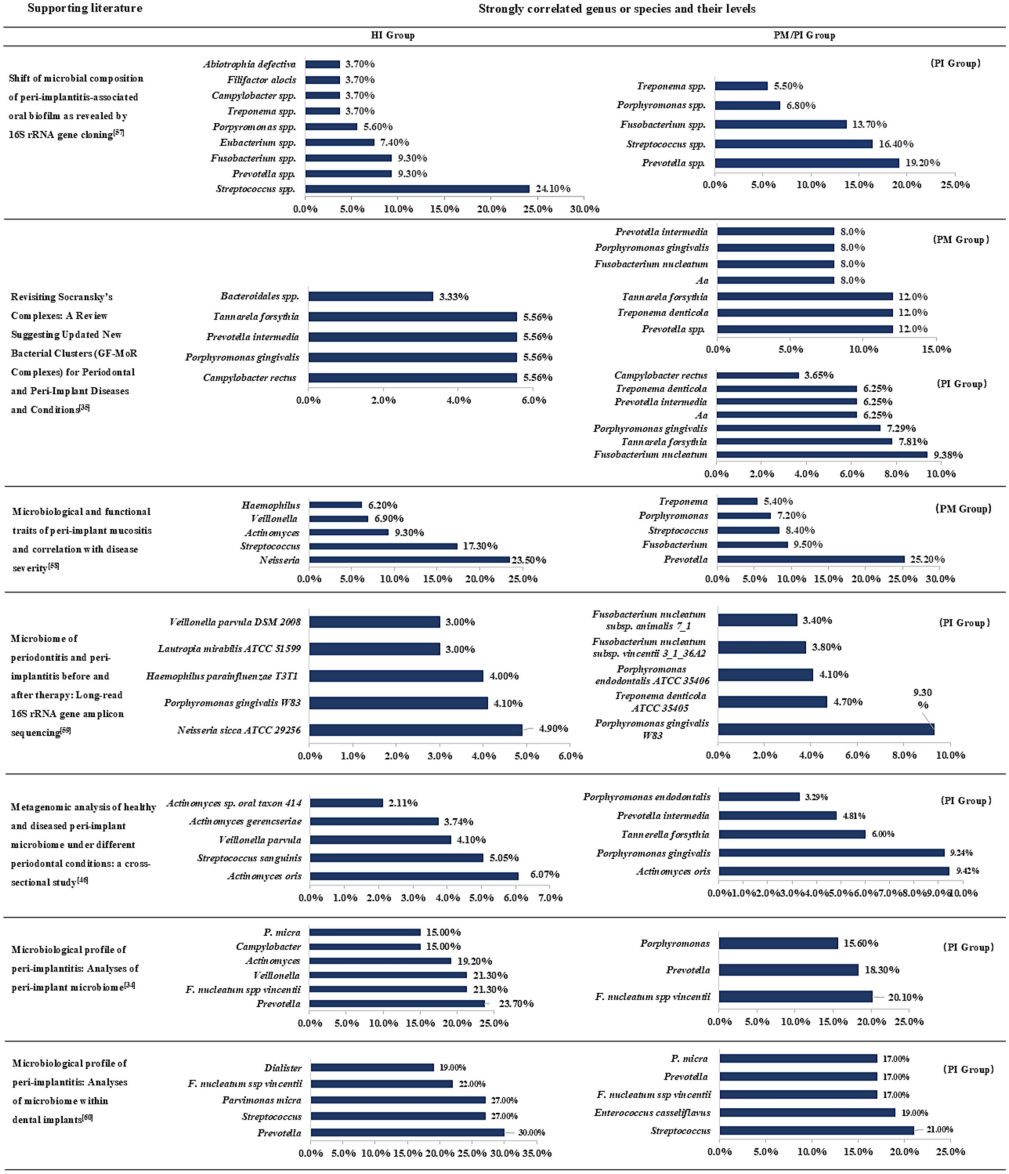
Subgingival microbiome in peri-implant health and disease states. PM, peri-implant mucositis; PI, peri-implantitis; Aa, Aggregatibacter actinomycetem-comitans. Combined with the data in different articles for comparative summary, this figure lists the top seven genus or species in each group and will indicate their groups.

In the above, we have summarized and discussed the types, mechanisms and clinical significance of several microbial complexes in order to enable clinicians to further understand the proportion of different bacteria in diseases and guide clinical practice. The following Figure 1 lists several high-quality studies in recent years that report the comparative status of subgingival flora classification and content in the peri-implant region in the healthy and diseased groups (Kensara et al., 2024; Fernandes et al., 2024; Song et al., 2024; Al-Ahmad et al., 2018; Feng et al., 2024; Yu et al., 2024; Kensara et al., 2023).

*Fretibacterium fastidiosum* and *Filifactor alocis* are unique pathogens found at peri-implant inflammation sites, corroborating the idea that the microecology of peri-implantitis overlaps with that of periodontitis but is more complex and variable. Furthermore, these pathogens may employ distinct mechanisms to induce peri-implant periodontitis (Sanz-Martin et al., 2017). *Monilia albican* is a fungal pathogen that can colonize and form biofilms in peri-implantitis. Notably, the biofilms formed by *Monilia albican* are very resistant to antimicrobial agents and host immunity, which in turn leads to disease progression and treatment difficulties (Pisano et al., 2023). An article on the analysis of subgingival fungal and bacterial diversity in patients with peri-implantitis showed that the interaction between the fungal and commensal bacterial flora leads to increased inflammation (Chen et al., 2024). Herpes simplex virus type I is a viral pathogen that infects epithelial cells, disrupts the epithelial barrier and lurks in the deeper biofilm layers, making it easier for bacteria to invade healthy tissues and leading to inflammation, suggesting a synergistic interaction between viruses and bacteria in peri-implantitis (Pérez-Chaparro et al., 2016). Human herpesvirus type IV is not a signature microorganism at peri-implantitis sites, but is still considered as a risk factor and peri-implantitis enhancer because its presence is positively correlated with peri-implantitis-associated pathogens (*T. forsythia* and *F. nucleatum*) (Canullo et al., 2018).

In the field of periodontology, a quantum leap in prevention, diagnosis, and treatment of the periodontal microbiota has been made since the publication of the Socransky complex. However, as our understanding of the complex interactions between the microbiota and the human body deepens, there is an urgent need to define and categorize the healthy oral microbiota, which is exacerbated by the popularity of modern dental implants. The characterization of normal microbial communities around healthy implants is an urgent issue that needs to be addressed in order to elucidate the role of microbiota dysbiosis in the development of peri-implant diseases, facilitate the development of implant dentistry and improve implant prognosis (Sun et al., 2023; Dutra et al., 2025).

### 3.3 Oral dysbiosis and immune system regulation

The immune system shapes normal or dysregulated micro-ecosystems and understanding its interactions with oral microorganisms is critical to unraveling the impact of host immunity in the development of peri-implant disease. The intrinsic immune system senses microorganisms through pattern recognition receptors, including Toll-like receptors (TLRs) (e.g., MyD88-associated TLRs, TLR5), NOD-like receptors (NLRs) (e.g., NOD1, NOD2), regulates oral microbial composition (Chu and Mazmanian, 2013). Inflammasomes formed by nucleotide-binding oligomerized structural domain-like receptor protein (NLRP) (e.g., NLRP6-associated) also maintain oral microbial stability (Ghimire et al., 2020). Inflammasomes (multiprotein complexes) are linked to certain endogenous danger signals that mediate dysregulated caspase-1 activation and promote IL-1β and IL-18 production (Franchi et al., 2009). A cross-sectional study showed that inflammasomes (AIM2, NLRP3), and their downstream effectors (interleukin-1β, caspase-1), are strongly associated with specific bacteria in peri-implantitis (Padial-Molina et al., 2024).

Antimicrobial peptides (from epithelial cells/innate lymphocytes) and adaptive immune components (including immunoglobulin A (IgA) secreted by B-cells, follicular helper T-cells, constant natural killer T-cells, and intra-epithelial lymphocytes expressing γδ T-cell receptor) maintain microbial homeostasis. Their aberrations are strongly associated with peri-implant diseases.

**Figure 2:**
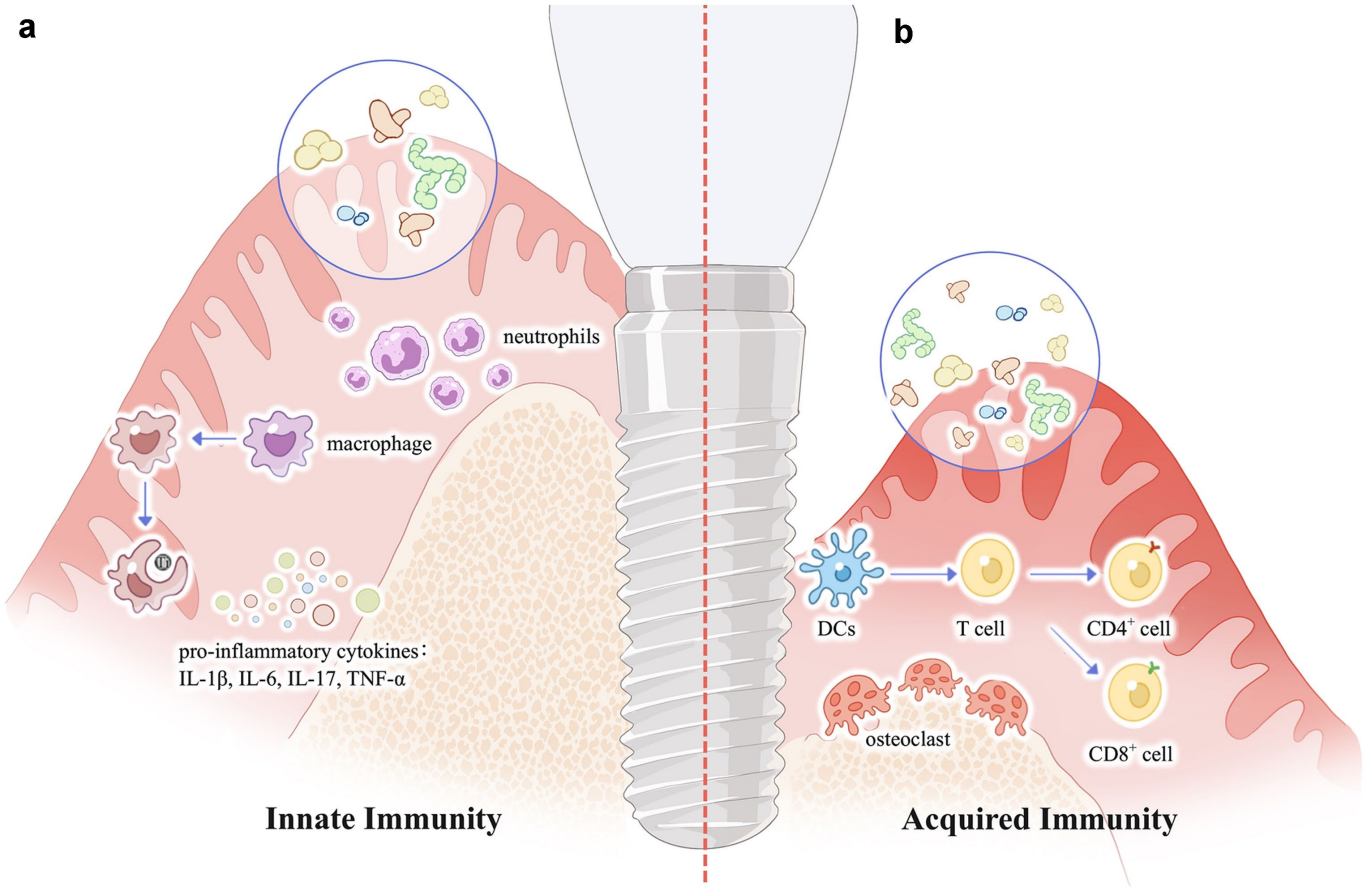
Schematic diagram of the mechanism of peri-implantitis caused by oral microecology. **(a)** Dysbiosis induces an innate immune response, with a large number of neutrophils infiltrating and macrophages polarizing, forming the M1 phenotype, and releasing pro-inflammatory cytokines. Vascular permeability increases, and the gums swell. **(b)** As the disease progresses, bacteria flood into the connective tissue, and immune cells such as CD4+, CD8 + gather, and differentiate into Th1, Th2, Th17 and regulatory T cells, etc., releasing pro-inflammatory cytokines IL-1β, IL-6, TNF-α, etc. Bone marrow mesenchymal stem cells differentiate into osteoclasts, leading to a large amount of alveolar bone absorption (Gasmi Benahmed et al., 2022; Checchi et al., 2020; Huang et al., 2024; Osta, 2014; Plemmenos, 2022; Abdulhameed and Omar, 2022).

Immune-microbiota crosstalk is bidirectional: Dysbiosis alters microbial molecular signatures (e.g., immunogenic lipopolysaccharides affecting TLR4, *P. gingivalis* degrading MyD88 or exploiting TLR2-C5aR), disrupting immune activation (Maekawa et al., 2014). This destabilizes epithelial barriers, triggers cell-mediated immunity, and amplifies inflammation (Figure 2). Dysbiosis also dysregulates microbiota-dependent pathways (e.g., NLRP6 inflammasomes, IL-22), perpetuating disease.

Implants, as foreign bodies, induce a macrophage-dominated inflammatory response. Osteoimmunology posits a delicate balance between bone resorption and formation. Dysbiosis disrupts this equilibrium: immune-derived pro-inflammatory factors (IL-1, TNF-*α*) propagate inflammation to alveolar bone, causing resorption and collateral damage (Abaricia et al., 2020). Macrophage polarization is pivotal—M1 macrophages exacerbate inflammation, while M2 macrophages promote osseointegration and healing (Wang et al., 2019; Pajarinen et al., 2013). An in-depth study of polarization dynamics at the implant-bone interface is thus crucial for integration success. In adaptive immunity, microbiota degrade secretory IgA (sIgA), altering ecological niches. Transfer of low-sIgA-adapted microbiota heightens host inflammatory susceptibility, underscoring microbes’ active role in reshaping oral ecology during dysbiosis.

### 3.4 Effects of host genetic factors (such as gene polymorphism) on dysbiosis

The host’s genetic background plays a key role in the composition and homeostasis of oral microbiome. Several studies have shown that polymorphisms in immunomodulatory genes (such as IL-1β, IL-6, TNF-α) can significantly affect the host’s immune response to oral microorganisms. A 2021 meta-analysis of 27 studies found IL-1α C-889 T, IL-1β C + 3,954 T, and IL-1β C-511 T are significantly associated with peri-implant disease. The composite IL-1 genotype showed nearly doubled risk (OR ≈ 1.95) of peri-implant disease (Jin et al., 2021). A meta-analysis (12 studies) found no overall association of TNF-α (−308 G > A) or IL-10 (−1,082, −819, −592) polymorphisms with peri-implant disease risk. However, TNF-α (−308 G > A) did show a significant risk increase (OR ~1.59) in Asian subpopulations (Jamshidy et al., 2021). A narrative review highlights polymorphisms in TNFα, MMP-8, IL-6, IL-1β. While biologically plausible, these show inconsistent or limited associations, often dependent on ethnicity, sample size (Chmielewski and Pilloni, 2023). Currently, no single genetic marker is robust enough for standalone clinical use due to heterogeneous evidence and confounding factors. Research on gene–environment interactions may improve risk stratification, but current guidelines prioritize behavioral and mechanical preventive measures (Wang et al., 2025).

### 3.5 Diagnosis and treatment of peri-implant diseases based on microdysbiosis

**Figure 3:**
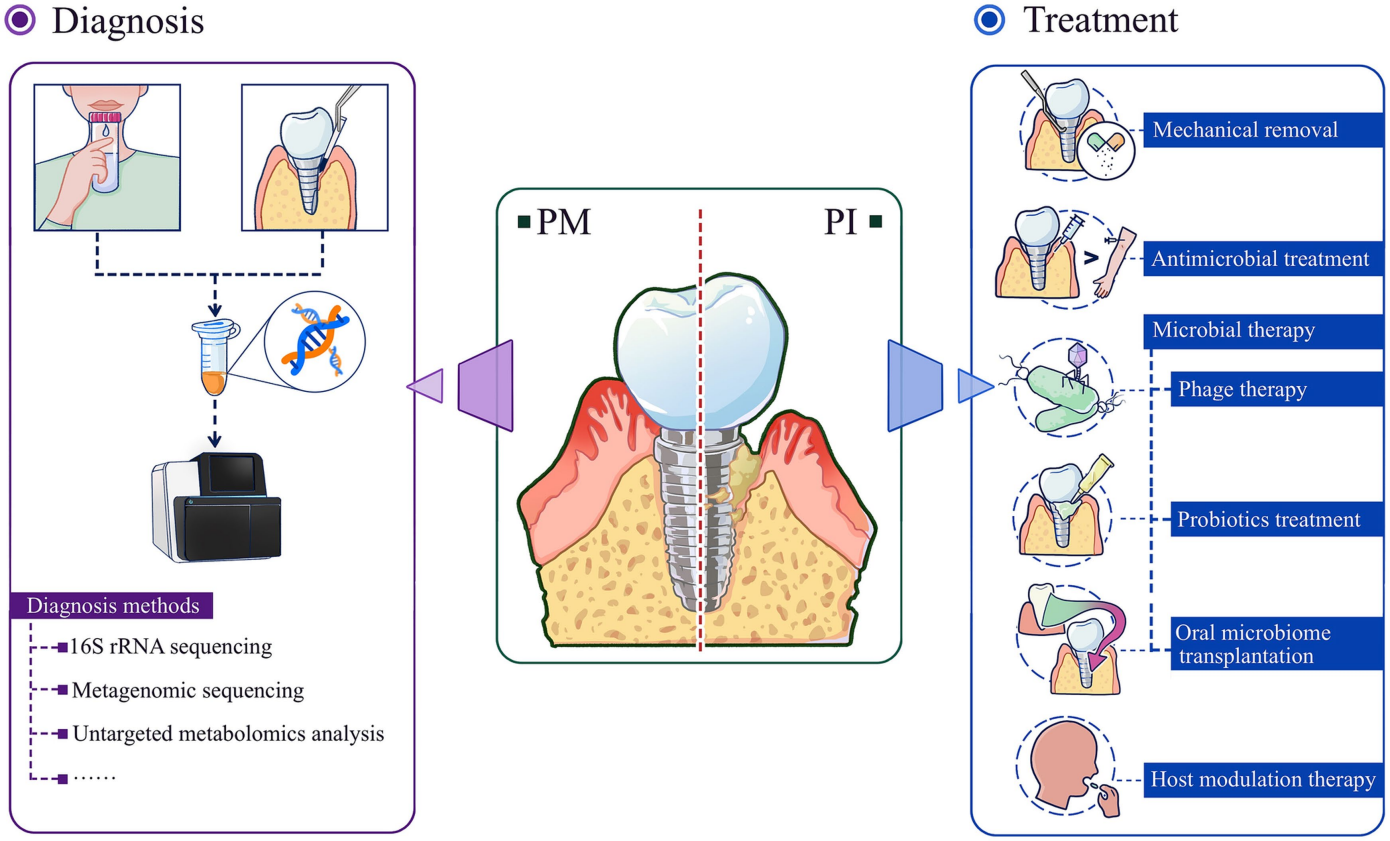
Diagnosis and treatment strategies of peri-implant diseases based on microdysbiosis. Advanced microbiome analysis tools are powerful means to prevent/diagnose peri-implant diseases; based on the microecological perspective, microbial therapies including phage therapy, probiotics treatment, oral microbiome transplantation are more effective and promising new therapies for the treatment of peri-implant diseases. PM, peri-implant mucositis; PI, peri-implantitis.

Similar to the progression of early periodontitis due to dysbiosis, peri-implant disease progresses through analogous microbial imbalances. By the time patients notice significant clinical symptoms, the condition has often progressed to mid- or late-stage. Inflammatory states are closely associated with an overall decrease in microbiota species richness and exacerbates disease-related microdysbiosis (Henson and Phalak, 2017). In the face of the complexity of multiple flora interactions, it is particularly important to rationally utilize bacterial detection techniques to their fullest extent. Similarly, based on the underlying logic of dysbiosis in peri-implant disease progression, treatment strategies need to be updated. Compared with traditional mechanical therapy and drug intervention therapy, microbial therapy is obviously more promising (Figure 3). It targets the bacterial hierarchy and aims to restore a balanced bacterial community ecology so that “healthy” microecology can help the peri-implant state “heal itself.”

#### 3.5.1 Diagnosis

Previous microbiological analysis of peri-implant disease relied on bacterial culture methods, which were limited by the inability to culture many oral microorganisms. Innovations in microbiome analysis tools, including DNA sequencing, RNA sequencing, and metagenomic sequencing for the identification of strains and their genomes, and untargeted metabolomic analysis, enable characterization of microbial community composition and function in peri-implant disease (Freire et al., 2021). These tools can overcome the limitations of previous analytical methods and help to identify microbial biomarkers that can provide early warning or strong diagnosis of dysbiosis and its preliminary stage (Chun Giok and Menon, 2023). By detecting microbial DNA in gingival sulcus fluid, it is possible to identify the species and number of pathogenic bacteria, providing more accurate information for diagnosis and treatment (Nazar Majeed et al., 2016). In addition, Matsuo et al. found that when studying *Clostridium difficile* and *Bifidobacterium bifidum*, two bacterial taxa with close relevance to the study of oral bacteria, full-length 16S rRNA gene sequencing could differentiate between the two with higher precision (Matsuo et al., 2021). Some bioinformatic tools such as 16S-ITGDB, a comprehensive database that enhances the classification of prokaryotic sequences, have improved the accuracy of identification of oral-associated bacteria (Hsieh et al., 2022).

#### 3.5.2 Treatment strategies

Measures related to the treatment of peri-implant diseases based on the microecological perspective include mechanical debridement, antimicrobial therapy, microbial-targeted therapy, and host modulation therapy. Among them, microbial targeted therapies include phage therapy, probiotic supplementation, and microbial transplantation. Host modulation therapies include non-steroidal anti-inflammatory drug therapy, bisphosphonate therapy, and pro-inflammatory cytokine antagonist therapy.

##### 3.5.2.1 Mechanical removal

Mechanical removal effectively removes plaque and tartar, which are the primary habitats for oral microorganisms. Mechanical debridement, a physical removal method, can significantly reduce the number of pathogenic bacteria, such as *Streptococcus pyogenes*, thereby reducing the risk of caries, periodontal disease, and peri-implant disease. However, this intervention may also lead to a transient imbalance in the oral microbial community. Some of the beneficial flora may also be removed during the debridement process, which may leave the oral microecology in an unstable state for a short period of time. Although mechanical debridement may temporarily disrupt the oral microecological balance, studies have shown that the oral microbial community is highly self-repairing. After debridement, the oral microbial community gradually returns to a relatively stable state. To accelerate this process, adjunctive treatments such as antimicrobial drugs or probiotics are often combined in clinical practice. For example, the use of emerging biomaterials such as antimicrobial peptides or nanomaterials can further inhibit the growth of harmful bacteria after debridement while promoting the recovery of beneficial bacteria. In addition, some studies have discussed in depth the mechanism by which arginine disrupts biofilm formation and reduces the effect of biofilm adhesion, thus serving as a potential method for preventing peri-implant disease (Gloag et al., 2021).

##### 3.5.2.2 Antimicrobial treatment

Topical adjunctive antibiotics can be used in the treatment of peri-implantitis. Systematic evaluations and meta-analyses have shown that topical application of antibiotics is beneficial in peri-implantitis and does not produce any adverse effects (Klinge et al., 2002; Lu et al., 2022). However, in many cases, topical application of antibiotics requires exposure of the implant surface and the bone defect. Case series and cohort studies have demonstrated the additional benefit of non-surgical treatment when systemic antibiotics are used as adjunctive therapy. However, the use of systemic antibiotics is controversial. Over-reliance on antibiotics may lead to problems such as bacterial resistance, gastrointestinal side effects, and dysbiosis. Therefore, clinicians usually recommend caution in cases where the infection is clearly severe or local treatment is ineffective, and strict guidelines for antibiotic use are followed (Klinge et al., 2012).

##### 3.5.2.3 Phage therapy

Phage therapies are a class of microbiome-based interventions that target bacteria involved in the pathogenesis of oral diseases with the aim of restoring the homeostatic function of the oral microbiota. Phages infect only bacteria and do not destroy eukaryotic cells and are therefore not toxic to humans (Grase et al., 2023). This property can address the increasing weakness of antibiotics in the face of bacterial resistance, and the small size of phages allows them to possess high penetration into biofilms. Phages are mainly categorized into lytic phage and temperate phages. Lytic phages are highly specific to the host, replicate in the host and lyse the host. Temperate phages integrate with the host DNA and multiply together, reversing the antibiotic resistance of the host and indirectly restoring the effectiveness of antibiotics. Current studies have found that different types of phages are highly specific for peri-implantitis-associated bacteria. φKSM96 temperate phages isolated from *Streptococcus pyogenes*, which significantly inhibited *Streptococcus pyogenes* growth and biofilm formation, and φKSM96 resulted in a significant decrease in the proportion of *Streptococcus pyogenes* in co-cultures of *Streptococcus pyogenes* with other bacterial species as reported by Sugai et al. (2023). *F. nucleatum* biofilms were disrupted by *F. nucleatum* phage FNU1 as observed under confocal microscopy in crystal violet staining experiments. Several lytic enzymes targeting *S. aureus* are currently at different stages of human research (São-José et al., 2022). The phenomenon of phage-antibiotic synergy has revealed that phages and antibiotics can be more effective at killing bacteria when they work together than when one is used alone, and possible mechanisms of action include phage enzymes breaking down bacterial polysaccharides to make antibiotics more effective (Łusiak-Szelachowska et al., 2022). Such mechanisms may provide ideas for eliminating specific bacteria in peri-implant disease. The existence of lysogenic phages that are highly specific for *P. gingivalis* has not been conclusively confirmed by research. With increasing evidence that phages are therapeutic, many regulatory agencies (e.g., U.S. Food and Drug Administration, etc.) are increasingly willing to approve the use of phages for therapeutic purposes for specific purposes (Kabwe et al., 2025). The rapid multiplication capacity of phages may result in extensive bacterial lysis and may release bacterial antigens, leading to abnormal immune responses in patients (Wei et al., 2024). The importance of the safety of phage therapy is therefore self-evident, and comprehensive *in vitro* cytotoxicity testing experiments on phages are currently inadequate. Phages used for the treatment of peri-implant diseases need to fulfill efficacy and safety requirements related to clinical trials before they can be approved. The large-scale application of phage therapy in peri-implant diseases presents several challenges, including regulatory issues, production scalability, phage-host specificity, and the potential for bacterial resistance. These challenges must be addressed to facilitate the widespread use of phage therapy in clinical settings (Sahoo and Meshram, 2024).

##### 3.5.2.4 Probiotics treatment

Probiotics fight disease through a variety of mechanisms. Colonization around the implant is the initial step in the therapeutic efficacy of probiotics. Probiotics exert an inhibitory effect on pathogenic bacteria through the production of antimicrobial substances, including bacteriocins, organic acids, short-chain fatty acids, and acetaldehyde (Piewngam et al., 2018). These inhibitory substances are effective in weakening the viability and metabolic activity of bacterial cells, e.g., *Streptococcus salivarius K12*, inhibits the biological activity of streptococci (Stašková et al., 2021). In addition, probiotics reduce pathogen-mucosal interactions by competing for binding sites on the epithelial surface, and they also compete with pathogens for nutrients. Probiotics exert immunomodulatory effects by coordinating T cell differentiation, stimulating IgA secretion, and promoting the production of anti-inflammatory cytokines (Suez et al., 2019). Probiotics also improve mucosal barrier function by inhibiting epithelial cell apoptosis and increasing the production of tight junction proteins. Prebiotics, as a class of functional foods, are not digested or absorbed by the host but can be selectively utilized by the body’s microorganisms, thereby promoting the body’s health. Prebiotics can selectively stimulate the growth of some beneficial microorganisms in the gut or increase the activity of these microorganisms to benefit the health of the organism. Currently, four clinical studies on the use of probiotics to treat peri-implant diseases have been completed, but none of them have yielded any results (Table 1).^1^ More research is needed in this area in the future.

**Table 1:** Clinical trials on probiotics therapy in peri-implant diseases.

NCT number	Study title	Study design	Conditions	Interventions	Primary outcome measures	Secondary outcome measures	Phases	Enrollment	Completion date
NCT05758103	*Limosilactobacillus reuteri* as an Adjuvant in the Treatment of Peri-implant Mucositis	Randomized, parallel, masking	Peri-implant mucositis	Experimental: Dietary supplement: *Limosilactobacillus reuteri* Prodentis^®^ (combining *L. reuteri* DSM 17938 and *L. reuteri* ATCC PTA 5289 strains) + Mechanical debridement; No Intervention: Mechanical debridement	Modified bleeding index, change of the bleeding score on each dental implant from baseline to 6 weeks and from baseline to 10 weeks	Modified plaque index, change of the bacterial plaque score on each dental implant from baseline to 6 weeks and from baseline to 10 weeks	NA	32	2022/9/18
NCT01974596	Use of Probiotics in Oral Health of Patients With Dental Implants	Randomized, crossover, triple masking	Mucositis	Two groups: with no peri-implant disease; with peri-implant mucositis. All participants will take probiotic tablets of *Lactobacillus reuteri*, wash up, then take placebo tablets.	Evidence in reduction of plaque index, the Mombelli classification	Evidence in reduction of bleeding around implants, reduction of probing depth, reduction interleukin 1β concentration, reduction interleukin 6 concentration, reduction interleukin 8 concentration	PHASE2	34	2010/7/1
NCT04187222	Effect of Probiotic Use *Bifidobacterium Animalis* Subsp. Lactis in Peri-implant Mucositis	Randomized, crossover, triple masking	Mucositis Oral	Experimental: Mechanical treatment + *Bifidobacterium animalis* subsp. Lactis; Control: Mechanical treatment + Placebo	Bleeding the probing, baseline, 12 weeks, 24 weeks		PHASE3	38	2019/11/15
NCT05921357	Effect of Fermented Products and Probiotics on the Condition of the Implant	Observational	Implant	Three groups: Peri-implantitis, Peri-implant mucositis, Peri-implant health; Conduct a questionnaire survey for all three groups.	Consumption frequency, consumption amount, daily intake, bleeding on probing, periodontal pocket depth	Plaque index, gingival index, clinical attachment level		126	2023/3/31

##### 3.5.2.5 Oral microbiome transplantation

In microbial transplantation, the fecal transplantation therapeutic approach aims to transplant the functional microbiota from healthy human feces, into the patient’s intestinal tract, to re-establish a new intestinal microbiota, and to achieve a microbiota regulation strategy for the prevention and treatment of intestinal and extraintestinal diseases (Lin et al., 2025). And in oral microecology, many concepts including periodontal microbial complex have been studied in many ways, can we assume that the model provided by the fecal colony transplantation theory is also applicable in the oral microecological environment? That is, oral microbiome transplantation (OMT): a promising approach to treat periodontal and peri-implant diseases by transferring the oral flora of a healthy donor to a recipient after minimal treatment, allowing the healthy flora to colonize the recipient’s oral environment and restoring the recipient’s oral microecology (Nascimento, 2017; Siddiqui et al., 2023). However, as some of the current challenges facing fecal colonization, there are some possible problems with oral colonization. First, the increasing complexity of oral microbiota, driven by modern dietary patterns and high prevalence of periodontitis (a significant proportion of implant patients will develop peri-implant inflammation) presents challenges. There is no precise definition of the structural characteristics of the flora in a healthy periodontal microecology. Secondly, we know that once peri-implant tissue related diseases develop, the biofilm is very difficult to remove, even with today’s more sophisticated periodontal treatments. Can the donor flora successfully occupy the space and colonize, changing the flora structure of the residual biofilm? Thirdly, from an immunological point of view, we still need to explore the survival mechanism of the donor flora in the recipient’s oral cavity. In addition, it is not yet known whether treating the implant surface with a healthy flora prevents inflammation of the peri-implant tissue. OMT involves the transfer of whole microbial ecosystems, which may harbor not only commensals but also opportunistic pathogens (Nascimento, 2017). In current stage, no standardized guidelines exist for OMT donor eligibility, microbial screening, processing or delivery protocols. This regulatory void presents safety liability, ethical and legal uncertainties, scientific reproducibility limitations (Nezhadi et al., 2024). Oral microbiota transplantation is still in the experimental stage. The clinical translation of oral microbiota transplantation requires interdisciplinary collaboration between microbiology, immunology, materials science, and clinical medicine, such as improving clinical evidence of safety and efficacy, developing standardized operating procedures, and analyzing microbial host interaction mechanisms. In conclusion, the microbial transplantation concept proposed by the flora transplantation treatment method provides a very broad idea and research space.

##### 3.5.2.6 Host modulation therapy

NSAIDs (NSAIDs) are one of the widely used drugs for the treatment of acute or chronic pain in oral diseases. They exert anti-inflammatory, analgesic and antipyretic effects through cyclooxygenase inhibition, thereby reducing the synthesis of prostaglandins. In oral and maxillofacial inflammation, NSAIDs can effectively relieve symptoms, such as pain and swelling, and improve the quality of life of patients. Their therapeutic and toxic effects have largely been demonstrated. Their combination of analgesic efficacy and fewer side effects than opioid drugs, justifies their prevalent use in oral medicine (Winnett et al., 2016).

However, the use of NSAIDs in the management of peri-implant disease is controversial. On the one hand, its anti-inflammatory effect may help control the inflammatory response of peri-implant tissues; on the other hand, as the inhibition of prostaglandins may interfere with normal bone metabolism and repair processes, thereby affecting the bone integration of implants. A retrospective study published by Etikala et al. (2019) found that the use of NSAIDs had a negative impact on the bone integration of titanium implants. Clinical studies have shown that short-term use of NSAIDs does not appear to negatively impact osseointegration, but long-term or high-dose use may increase the risk of implant failure (Kumchai et al., 2021).

Bisphosphonates are a class of pyrophosphate analogues that can selectively adsorb to the bone mineralization matrix. Upon uptake by osteoclasts, they inhibit osteoclast activity and induce apoptosis, thereby suppressing bone resorption. In addition, bisphosphonates exhibit anti-inflammatory properties and the ability to inhibit bacterial biofilm formation. Meraw et al. found that topical application of alendronate significantly increased the rate of osteogenesis in dogs with a model of peri-implantitis (Łusiak-Szelachowska et al., 2022). Another systemic review including 378 patients shows better results in some cases for dental implant therapy in cases of bisphosphonate intake (Fiorillo et al., 2022). A meta-analysis published by Lin et al. (2025) included 21 studies and found that the use of bisphosphonates may be associated with implant failure. Further high-quality studies are necessary to clarify their therapeutic potential and safety profile.

Pro-inflammatory cytokine antagonists can effectively block the inflammatory response and bone resorption process of peri-implantitis by specifically inhibiting the activity of key inflammatory mediators such as IL-1β and tumor necrosis factor-*α*. *In vivo* studies have shown that periodontitis patients treated with anti-tumor necrosis factor-α significantly reduced IL-1β and IL-8 levels in gingival sulcus fluid and IL-8 levels in saliva (Kabwe et al., 2025). The advantages and disadvantages of different treatment strategies are summarized in the following Table 2 (Meraw et al., 1999).

**Table 2:** Summary of different treatment strategies for peri-implant diseases (Freire et al., 2021; Klinge et al., 2002; Klinge et al., 2012; Ustun et al., 2013).

Treatment strategy	Strengths	Weaknesses
Mechanical removal	Effective in removing plaque and tartar. Less trauma, faster patient recovery and less postoperative discomfort. Simple to operate and widely used clinically. Can be performed multiple times, suitable for long-term maintenance treatment.	Difficult to completely remove all plaque and tartar, especially deep infections. Improper handling may damage the implant and affect its long-term stability. Unable to restore bone tissue, need to be combined with other treatments.
Antimicrobial treatment	Directly inhibit or kill disease-causing microorganisms to reduce infection. Combined with mechanical debridement, it can improve the therapeutic efficacy, especially for deep infection. Effectively reduce the inflammatory response, alleviate the symptoms, and promote the healing of the tissues. Convenient to use, with a high degree of patient compliance. Regular use of antimicrobials can help to prevent the disease from recurring, and to maintain the health of the peri-implant area.	Long-term or inappropriate use of antimicrobial drugs may lead to the development of drug-resistant strains of bacteria and reduce the effectiveness of treatment. Systemic use of antibiotics may cause gastrointestinal reactions, allergies and other side effects; topical use of antibiotics may lead to dysbiosis in the oral cavity. Inability to remove plaque and tartar, which needs to be combined with mechanical removal. Higher cost. Dependent on patient compliance.
Microbial-targeted therapy	Characterized by precision and personalized treatment. Innovative using cutting-edge technologies.	Technically complex and difficult to implement. Off-target effects. Effects vary from person to person, lack of uniform standards.
Host modulation therapy	Controls disease progression by modulating the body’s immune response and reducing excessive inflammatory responses. Promotes tissue repair and regeneration. Improves overall therapeutic efficacy when used in conjunction with mechanical clearance and antimicrobial therapy. Targets the underlying causes of disease, not just the symptoms, contributing to long-term disease control. By modulating the host response, it can reduce reliance on antibiotics and reduce the risk of drug resistance.	Efficacy may vary according to individual differences, and some patients may respond poorly. Some drugs may cause gastrointestinal discomfort, immunosuppression, and other side effects. Higher cost. Need for individualized treatment, which increases the complexity of treatment. The safety and efficacy of long-term application need further study, and there may be unknown risks.

## 4 Future research directions and challenges

The literature related to peri-implant diseases in recent years is still at the stage of defining, understanding, and drawing analogies for dysbiosis, and there are few papers with a large amount of data to support as research evidence. Some new points of association have been identified in the literature to inspire new diagnostic and therapeutic approaches in the future, but there are still many challenges that need to be overcome for the field to fully utilize the new information about the different states of the microbial ecosystem and their role in disease development. Moreover, sample sizes in the published literature are generally small. In addition, differences in sampling techniques, laboratory contamination, and selection of patient populations have led to widely variable results.

The direction of research on peri-implant diseases will be multidimensional and interdisciplinary. First, the development of new microecological regulation methods will become a research hotspot. With the continuous progress of microbiomics technology, the methods of precise identification and regulation of peri-implant microbial communities will be further developed. For example, novel therapeutic strategies based on probiotics, phage therapy, or microbial metabolite modulation are expected to prevent and treat peri-implantitis by restoring microecological balance. Second, future studies should focus on large-sample, multicenter research while adopting standardized sampling and stratified analysis of patients to explore the causal relationship between microecological dysregulation and peri-implant disease (Liu et al., 2024). In addition, the introduction of artificial intelligence and machine learning technologies will promote the development of personalized diagnostic and treatment plans, and early prediction and intervention of disease risk will be achieved through the in-depth analysis of massive clinical data. Finally, interdisciplinary collaboration will accelerate the development of novel biomaterials, such as coating materials with antibacterial, anti-inflammatory, and osseointegration-promoting functions, to provide new solutions for the prevention and treatment of peri-implant diseases. In summary, future research will comprehensively improve the prevention and treatment of peri-implant diseases through a combination of technological innovation and clinical validation.

## Footnotes

^1^Available online at: https://clinicaltrials.gov (accessed June 27, 2025)
